# Globalization in the Healthcare Industry: Drivers, Risks, and Adaptation

**DOI:** 10.3390/healthcare14091177

**Published:** 2026-04-28

**Authors:** Anasztázia Kész, Ildikó Balatoni

**Affiliations:** 1 Doctoral School of Management and Business, University of Debrecen, 4032 Debrecen, Hungary; kesz.anasztazia@med.unideb.hu; 2 Clinical Center, University of Debrecen, 4032 Debrecen, Hungary

**Keywords:** globalization, healthcare industry, artificial intelligence, supply chains, ageing society, climate change, health-conscious lifestyle, biotechnology, digital health

## Abstract

Globalization refers to the increasing density of economic, social, and technological interconnections on a global scale. In the healthcare industry, it simultaneously accelerates innovation and increases systemic vulnerabilities. This study aims to review and conceptually synthesise the main channels of impact: (1) pharmaceuticals, clinical development, and regulation; (2) supply chains and resilience; (3) service mobility (health tourism); (4) human resources and competencies; (5) digitalization, artificial intelligence (AI), and data governance; (6) ethics, law, and public policy; and (7) sustainability and climate change. The COVID-19 pandemic highlighted the risks associated with global interdependencies, particularly in supply chains, while also demonstrating the innovation-accelerating effects of knowledge sharing and international cooperation. Particular attention is given to artificial intelligence and digital health, which open up new potential for efficiency and quality improvement from research and development through diagnostics to healthcare organization, while simultaneously intensifying concerns related to data protection, cyber security, and liability. Telemedicine, platform-based systems, and real-world data may contribute to addressing the care needs of ageing societies, but only when supported by appropriate competencies and sound data governance. As global data flows intensify, the importance of data protection, bias mitigation, transparency, and accountability correspondingly increases. Through the cultural channels of globalization, health-conscious lifestyles and complementary approaches are also spreading, which we address in a brief, separate subsection. The guidelines of international organizations foster standardization; however, due to differences in local capacities and institutional environments, the effects are not homogeneous. In conclusion, the study emphasises the dual nature of globalization; it expands access and accelerates innovation, while at the same time creating new vulnerabilities—in supply chains, labour mobility, and data security—and, together with climate-related risks, generating complex adaptive pressures for the healthcare industry.

## 1. Introduction

As a result of globalization, manufacturing and service companies must pay increasing attention to improving efficiency, reducing costs, and optimising operations in order to remain competitive in a rapidly changing market environment [1]. A distinctive feature of the healthcare industry is that competitive pressure coexists with the public interest (access, equity, and patient safety); consequently, the effects of globalization in this field give rise to particularly complex implications.

In this review, globalization is understood as the growing cross-border interconnectedness of goods, services, people, knowledge, technologies, data, and regulatory influences [2]. The term healthcare industry is used here in a broad sense to include the development, delivery, organization, and governance of healthcare, while health systems refers more specifically to the institutional arrangements through which healthcare services are financed and provided [3].

These processes also have a significant impact on healthcare. Globalization is reshaping the operation of healthcare systems. The guidelines of international actors (e.g., WHO, the UN, and the World Bank), together with their financing and standardisation mechanisms, influence regulation, access, and quality assurance. The liberalisation of international trade and technological development facilitate faster access to new medicines and treatment methods, while also increasing interdependence among healthcare systems [2]. At the same time, globalization also presents challenges for healthcare. Through global trade and travel, epidemics may spread more rapidly, while disruptions in supply chains—e.g., shortages of raw materials and transport problems—highlight the vulnerability of the system [4]. The COVID-19 pandemic, in particular, demonstrated that global dependencies (raw materials, manufacturing capacities, and logistics) may cause delays and inequalities in access even in the case of essential products [4]. However, the flow of information and the global coordination of resources may have contributed to the development and distribution of vaccines. The development and approval process of mRNA vaccines against COVID-19 reached widespread application in less than one year, with previous platform developments in mRNA technology providing important enabling conditions [5]. The aim of this study is to organize and synthesise the effects of globalization on the healthcare industry along major channels of impact, highlighting the sectoral implications of innovation and regulation, supply chains, and service mobility, as well as issues related to human resources and competencies, and the interconnections among digitalization, artificial intelligence (AI), data governance, ethics, law, public policy, and sustainability. This review responds to the need for more integrative synthesis in a field where the effects of globalization on healthcare are often discussed across separate thematic domains. The theoretical premise of the review is that the effects of globalization on healthcare should be understood not as a series of isolated topics, but as a system of interrelated channels of impact. Accordingly, the article discusses the main channels of impact and, in its conclusions, emphasises that the effects of globalization are not homogeneous; under differing national and regional capacities, institutional arrangements, and social contexts, different outcomes may be expected. The review may be relevant for researchers, decision-makers, and policy audiences concerned with globalization and healthcare. Following a methodological overview, subsequent sections analyse the principal channels of impact, while the conclusions synthesize these findings within a unified conceptual framework.

## 2. Materials and Methods

This study is based on a narrative literature review, which maps the effects of globalization on the healthcare industry along major channels of impact and then organizes the main consequences within a conceptual synthesis framework. Data collection was carried out in the form of a literature search using peer-reviewed scientific databases such as PubMed, Scopus, Web of Science, and Google Scholar. The main thematic blocks included globalization and health, the pharmaceutical industry, digitalization and artificial intelligence, health workforce issues, medical tourism, health system access, and global processes such as pandemics, COVID-19, and supply chains. Representative Boolean search strings included combinations such as (“globalization” OR “globalisation” OR “global health”) AND (“healthcare industry” OR “healthcare system” OR “health systems”); (“pharmaceutical industry” OR “drug development” OR “global clinical trials” OR “multi-regional clinical trials”) AND (regulation OR ethics); (“health supply chains” OR “pharmaceutical supply chains” OR “supply chain”) AND (resilience OR shortages OR vulnerability); and (“digital health” OR “artificial intelligence” OR “clinical decision support”) AND healthcare.

**Figure 1:**
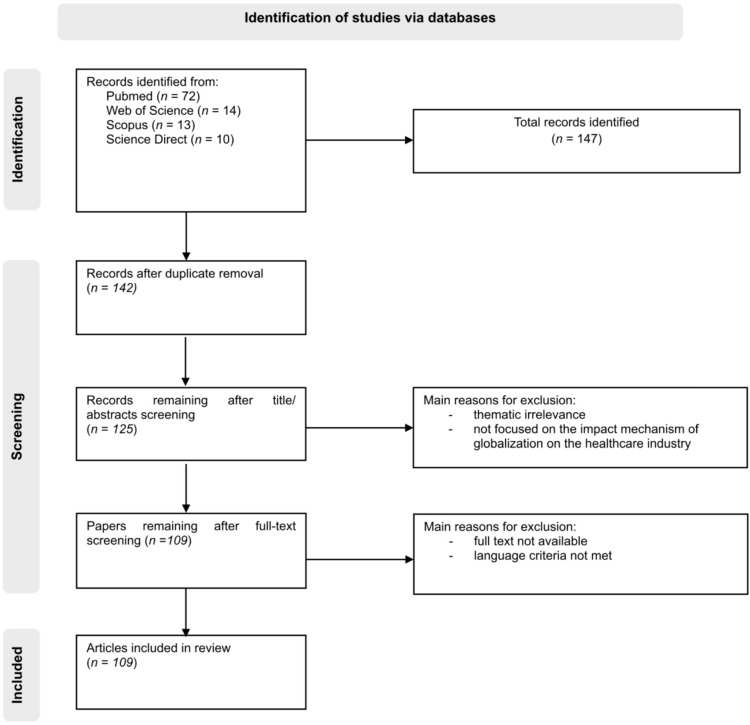
PRISMA-like flow diagram of the search and selection process for the narrative review.

The Complementary and Alternative Medicine (CAM) topic was included only as a supplementary, separate block in the search. The criteria for selecting studies were year of publication (2000–2025), language (English or Hungarian), availability of full text, and relevance to the themes of globalization, technological integration, industry operation, and the policy-regulatory environment. In this review, relevance was assessed in relation to the predefined channels of globalization and the healthcare industry outcomes examined in the study. The selected sources were screened and organized thematically within a narrative review design. Screening and inclusion/exclusion decisions were made on the basis of the predefined eligibility criteria and thematic relevance to the study framework. The literature search was conducted between December 2024 and February 2025. A total of 147 records were identified across the four databases. After the removal of duplicates, 142 records remained. Following title and abstract screening, 125 articles were assessed in full text, of which 109 sources were included in the narrative synthesis. The main reasons for exclusion were thematic irrelevance, lack of full-text availability, failure to meet the language criteria, and insufficient focus on the mechanisms through which globalization affects the healthcare industry. The search and selection process is illustrated in Figure 1. No formal meta-analysis or standardized quality appraisal protocol was applied, as the aim of the study was to identify the conceptual map and the main mechanisms of impact. At the same time, in order to mitigate interpretive bias, priority was given to systematic reviews, guidelines issued by international organizations, and well-established empirical studies. Older seminal sources were retained where they provide conceptual foundations, while more recent references were used to reflect current developments and empirical evidence.

For the synthesis, an “impact channel–consequence” matrix was developed: the rows contained the main channels of globalization (R&D and regulation; supply chains; service mobility; human resources; digitalization/AI/data; ethics, law, and public policy; sustainability/climate; cultural channels/CAM), while the columns represented industry outcomes (access, cost/efficiency, quality and patient safety, equity, resilience, innovation dynamics, and risks). During the review of the sources, a thematic and context-sensitive approach was adopted by identifying recurring patterns and points of connection. To improve the transparency of the conceptual synthesis, Table 1 presents the impact channel–consequence matrix that guided the organization of the reviewed literature and shows how the main channels of globalization relate to key healthcare industry outcomes. The steps of the conceptual synthesis were as follows: (1) extraction of relevant claims and empirical examples; (2) placement within the matrix (channel × outcome); (3) identification of consistent patterns and contradictions; and (4) formulation of chapter-level conclusions, with particular emphasis on non-homogeneity (differences by country/region). The matrix was intended as a conceptual synthesis tool rather than a quantitative evaluation framework.

The purpose of the approach was coverage and conceptual clarification. Owing to the heterogeneity of the literature, causal claims are generally formulated in cautious, indicative language, and no attempt was made to provide a quantitative synthesis.

## 3. Mechanisms of Impact and Industry Consequences

The effects of globalization on the healthcare industry operate through several interrelated mechanisms: (1) pharmaceutical industry, clinical development, and regulation; (2) supply chains and resilience; and (3) service mobility (health tourism). The aim of this chapter is to organize these channels in terms of their industry consequences (access, innovation, cost/efficiency, patient safety, equity, and risks).

### 3.1. Pharmaceuticals, Clinical Development, and Regulation

The pharmaceutical industry is one of the fundamental pillars of the global healthcare industry. Beyond its economic significance, it directly influences access, patient safety, and public health outcomes. It plays a key role in the research and development of new medicines, as well as in shaping the health policy frameworks governing their use. In addition, it is essential that the industry actively contributes to ensuring that patients have timely and equitable access to treatments based on the latest scientific advances [6]. The pharmaceutical industry is facing growing challenges in the development of effective and rapid drug discovery methods, which makes the introduction of new technological solutions increasingly urgent [7]. Technological innovations and digitalization have brought about significant changes not only in healthcare delivery, but also in pharmaceutical research and biotechnology, opening up new dimensions in both fields [8].

Drug discovery is, in itself, an extremely costly and high-risk process. It may take as long as 10–15 years, and development costs often reach USD 1–2 billion [9]. The application of artificial intelligence (AI) is transforming this field by enabling the faster and more targeted identification of potential drug candidates, thereby generating substantial savings in both time and cost [10,11]. In small-molecule drug design, computational simulations already serve to screen promising compounds at an early stage [12]. This is particularly important because a substantial proportion of drug candidates fail during clinical trials [13]. In clinical development, particularly in Phases II and III, the role of AI and analytics may lie primarily in supporting patient selection, risk and outcome prediction, and trial operations (including recruitment and data quality), thereby indirectly improving efficiency and reducing delays. Generative AI approaches appear promising in the design and optimization of novel molecules; however, their clinical utility depends on transparent validation and regulatory acceptability [11,14].

#### 3.1.1. The Globalization of Clinical Trials and Its Ethical Implications

The opportunities and potential benefits offered by international clinical trials and regulatory approval strategies are increasingly taking on a global dimension [15]. Over the past two decades, the nature of clinical research has undergone a fundamental transformation. While it was previously concentrated primarily in developed countries, it has now become increasingly global, involving less developed regions of the world as well [16]. In parallel with the globalization of pharmaceutical development, multinational pharmaceutical companies are increasingly conducting multi-regional clinical trials (MRCTs), with the aim of bringing medicines to market more rapidly and ensuring broader patient access to innovative treatments [17]. The geographical scope of clinical trials has expanded considerably, and many more countries have become involved in international research networks. At the same time, the importance of quality assurance, ethical compliance, and data integrity has increased [16,18]. In this context, prospective registration in publicly accessible trial registries, adherence to contemporary data integrity standards, and international oversight practices have become increasingly important governance mechanisms [18,19,20]. Preserving and enhancing the competitiveness of clinical trial sites is in the shared interest of patients, trial centres, and sponsors alike. For patients, such trials provide an opportunity for early access to innovative therapies, while for trial centres, clinical research may become an integral part of care provision alongside knowledge development and the accumulation of experience [21].

In recent years, multinational pharmaceutical companies have increasingly conducted clinical trials in low- and middle-income countries, where regulatory environments are often less stringent and costs are lower. However, this trend raises a number of ethical concerns, particularly with regard to informed consent and the protection of participants’ rights. The Abdullahi v. Pfizer case, for example, highlighted that the absence of adequate information and consent may have serious consequences, thereby reinforcing the need for stricter ethical standards and legal mechanisms [22]. Under pressure from international human rights organizations and the media, United States courts have also assumed an increasingly important role in holding multinational pharmaceutical companies accountable. The Alien Tort Statute (ATS), for example, provides an opportunity for foreign citizens to seek legal remedy through United States courts in cases of human rights violations linked to the activities of multinational corporations [23,24]. The expansion of global clinical research makes it necessary to strengthen ethical standards and legal mechanisms in order to ensure the protection of participants’ rights and the integrity of research. International cooperation and improvements in the timeliness of regulatory and ethical approvals are essential for ensuring responsible and ethical pharmaceutical development [18].

#### 3.1.2. Regulation, Access, and “Value Demonstration”

Regulatory frameworks established by the WHO—such as the International Health Regulations (IHR)—may provide a more uniform framework for responses to epidemics and, more broadly, support international coordination [25,26]. In addition, initiatives such as the WHO Essential Medicines List and global pharmaceutical supply programmes may help to improve access in poorer countries as well [26]. Alongside these developments, the results of clinical trials now play a key role not only from scientific and ethical perspectives, but also from an economic one. In addition to the traditional expectations of safety, efficacy, and quality, pharmaceutical companies are increasingly required to demonstrate the economic value of their products for reimbursement and funding decisions, which creates new challenges for development and market strategies [27]. Overall, the globalization of the pharmaceutical industry and clinical development may simultaneously accelerate innovation (through international flows of knowledge and data, MRCTs, and digital tools) and increase risks (ethical exposure, unequal access, and regulatory heterogeneity). Technological breakthroughs—such as artificial intelligence and computer-aided drug design—may become a sustainable advantage at the industry level only if cooperation among academia, industry, and regulatory bodies deepens, and if ethical, data protection, and access-related considerations are placed within manageable frameworks [8,14].

### 3.2. Supply Chains and Resilience

The resilience of global supply chains is of critical importance, particularly during pandemics and crisis situations. Digitalization and data-driven supply management may improve visibility, inventory management, and distribution decisions, but they cannot replace the role of physical capacities and supplier diversification. Global supply chains underwent significant transformation as a result of the coronavirus pandemic, highlighting the problems of raw material shortages and excessive import dependence [28]. The vulnerability of pre-pandemic long supply chains, built on international division of labour and cost efficiency, became evident, particularly in industries operating with minimal safety stocks [29]. In healthcare systems, this was especially apparent, as shortages of raw materials and import dependence resulted in severe supply disruptions, for example in relation to medicines and medical devices. The disruptions caused by the pandemic underscored the importance of security of supply and resilience, while processes of deglobalization and the growing prominence of regionalization created new challenges and opportunities [28]. These vulnerabilities were not limited to pandemic conditions: shortages had already been increasing prior to COVID-19, particularly among older off-patent medicines and certain medical devices, while more recent evidence also points to structural concentration in upstream pharmaceutical supply chains and dependence on a limited number of manufacturing sites [30,31].

The logistical challenges arising from the production and distribution of vaccines required particular attention. During the COVID-19 pandemic, vaccine distribution represented an unprecedented logistical task, as several billion doses had to be delivered worldwide. Different types of vaccines—such as gene-based mRNA vaccines, vector-based vaccines, and vaccines containing inactivated pathogens—required different storage and transport conditions, which further increased the pressure on the logistical system [32]. These experiences made visible the strategic importance of the cold chain, last-mile distribution, and global capacity bottlenecks. According to the WHO, during the pandemic healthcare systems widely experienced disruptions in primary care, in the supply of medicines and vaccines, and in the movement of international health commodities [33]. Global inequalities in vaccine distribution highlighted deeper structural deficiencies in supply chains, particularly in low-income countries. The COVID-19 pandemic made these inequalities particularly visible: more broadly, it interrupted the previous decline in global inequality in 2020 [34], while within the health sector full COVID-19 vaccination coverage among health and care workers by the end of 2021 was 33% in low-income countries, 83% in lower-middle-income countries, 79% in upper-middle-income countries, and 88% in high-income countries [35]. The United Nations Conference on Trade and Development (UNCTAD) also emphasised that trade in healthcare and medical technology products surged during the pandemic, while some countries introduced export restrictions, which may in the short term have further exacerbated shortages [36].

All of this made new approaches to the design of logistical systems necessary, highlighting the role of localization, strategic stockpiling, and rapid response capability. In response, companies began to redesign their supply chains, by including more regional forms of cooperation and procurement models relying on multiple suppliers; furthermore, targeted inventory strategies emerged in order to mitigate future disruptions [28]. At the same time, the resilience agenda is not merely technological, and it also requires institutional coordination, risk sharing, and regulatory learning, particularly in times of crisis. In summary, the globalization of supply chains simultaneously creates economies-of-scale advantages and new vulnerabilities. Increasing resilience therefore requires a combination of supplier diversification, strategic stockpiling, regional capacities, and data-driven supply management [28].

### 3.3. Service Mobility: Health Tourism

Health tourism (cross-border patient mobility) represents the service-side channel of globalization. Patients choose between countries on the basis of cost, waiting time, specialized care, or quality preferences, while also influencing the financing, capacities, and regulation of both receiving and sending systems [37,38]. The literature focuses on those consumers who choose foreign or cross-border destinations for the purpose of obtaining healthcare, for example in the case of cosmetic, dental, cardiological, orthopaedic, or reproductive treatments [37]. Digital platforms and information channels have also intensified decision-making processes and competition among providers [39]. As cross-border treatments have become easily accessible with the spread of technology, it must be recognized that health tourism is not a homogeneous industry. Businesses operate with diverse business models and service profiles, not all of which fit the classic “tourism–treatment” scheme [40]. It is not only countries with advanced healthcare systems that attract foreign patients. Many people also travel to other countries for more affordable, more rapidly available, or specialized treatments [38]. Along certain social, political, and legal constraints, cross-border healthcare mobility may also be interpreted as a form of compelled action [41]. Within the EU, patient mobility is also shaped by the frameworks governing cross-border healthcare and by regional relations. In the case of Hungary, relations with neighbouring countries may likewise influence the direction and volume of these flows [42]. Since health tourism is bilateral in nature—involving both patients and providers—it is essential to examine how healthcare systems may be structured so as to address challenges related to quality assurance, financing, and ethics [43,44]. At the same time, increasing mobility may also entail public health risks: the spread of infectious diseases and issues of continuity of care (follow-up, documentation, and responsibility) become particularly salient [45]. Health tourism therefore represents both a market opportunity and a regulatory and public health challenge, to which targeted solutions in quality assurance, data sharing, and patients’ rights should be developed [44].

## 4. Human Resources and Competencies

In the globalized healthcare industry, workforce shortages, migration, the growing care demands of ageing societies, and the rise of digitalization and data-driven operations simultaneously increase both system pressures and competency requirements.

### 4.1. Workforce Mobility

The migration of healthcare professionals, one of the major consequences of globalization, threatens the stability of healthcare systems worldwide [46], while the place of training plays a decisive role in shaping migration rates [47]. Patterns of healthcare mobility may also differ by specialty and career stage, reflecting different responses to regulation and to demand–supply conditions [48]. The effects of global healthcare workforce migration strongly affect not only destination countries but also source countries, as the outmigration of highly skilled professionals from low-income countries may further exacerbate problems relating to the quality and availability of care [49]. The migration of skilled labour is driven primarily by better living conditions, professional opportunities, the desire for advancement, and the prospect of advantages offered by developed countries, forming part of a long-standing global trend [50].

The consequences of workforce flows are twofold. Destination countries may gain capacity in the short term, whereas in sending countries “brain drain” may increase waiting lists, territorial inequalities, and the risk of burnout [49]. This asymmetry is further supported by the study of Boniol et al., which reported that in 2020 the density of health workers in high-income countries was 6.5 times that of low-income countries [51]. Consistent with this, a South African trend analysis found that in 2017, 21.6% of South African physicians had active registration in destination countries, suggesting that outward mobility may also have a lasting effect on the capacities of upper-middle-income countries [52]. At this point, globalization is not merely a labour market phenomenon, but also an issue of equity and systemic resilience. In response to the inequalities caused by the migration of healthcare workers, global governance systems have emerged, incorporating various codes of practice, agreements, and reporting requirements [53]. For example, the WHO Global Code plays a key role in framing sustainable workforce policies, but because of its voluntary nature its effectiveness is limited, and consequently, strengthening implementation and data-reporting capacities is necessary in order to mitigate this trend [54,55]. Accordingly, the focus of policy responses is shifting increasingly towards retention, the improvement of working conditions, and the enforcement of ethical recruitment principles [53,54].

In this context, the decolonisation of globalized healthcare systems also becomes relevant, i.e., the recognition that the asymmetrical distribution of knowledge, human resources, and decision-making often operates to the advantage of countries in the Global North. A decolonial approach encourages developed states not merely to remain beneficiaries of global healthcare workforce flows, but also to contribute actively to strengthening local systems—for example through educational support, bilateral cooperation, or adherence to ethical recruitment guidelines [56]. The application of the root–stem model may help policymakers to address healthcare worker brain drain in a more structured manner, particularly in low-income countries [56]. This perspective is essential if global healthcare workforce mobility is not to deepen existing injustices further, but instead to promote the development of a fairer and more sustainable international healthcare system. Effective workforce planning and rigorous reporting systems may ensure the availability of appropriate data, thereby enabling the necessary decisions to be taken [53]. In addition, for countries seeking greater self-sufficiency, international cooperation and a fairer distribution of the healthcare workforce are of particular importance in reducing the disparities between high- and low-income countries [53].

The issue of workforce mobility is also closely linked to demographic trends. The growing care needs of ageing societies intensify workforce shortages and increase the importance of developing age-friendly health systems (AFHS) [57]. Pressure on demand and tensions in labour supply both encourage task shifting, teamwork, and—where feasible—digital support, but these can function effectively only if retention and training capacities are strengthened in parallel. In summary, global healthcare workforce migration represents a multi-level governance challenge. It simultaneously affects dimensions of equity, capacity, and sustainability, and therefore policy responses must increasingly be based not on recruitment alone, but on a combination of retention, training, ethical international cooperation, and data-driven planning [53].

### 4.2. Education and Competencies

Internationalization is fundamentally shaping the future of medical education, creating new challenges and opportunities. Global healthcare delivery, international mobility, and the development of educational standards all play a role in the evolution of medical education. Today, medical universities worldwide are characterized by diversity and by the continuous development of curricula [58]. Future directions point towards transnational approaches, in which internationalization is embedded in the curriculum and built upon cooperation among different countries [58]. The continuous development and transformation of medical education make it necessary to rethink the roles of teachers and students, as well as to improve the quality of learning processes. Effective curricula and educational principles are essential to ensure that future physicians are able to meet the demands of a rapidly changing medical and scientific environment [59]. At the same time, alongside globalization, there is a growing need not only for clinical knowledge but also for transferable competencies, including digital skills, data interpretation, interprofessional collaboration, quality improvement, and intercultural communication.

#### 4.2.1. Competency-Based Education, Generalism, and the Development of Practical Skills

Generalism, that is, broad medical knowledge and a systems-based perspective, is essential for ensuring effective care, as modern physicians must be competent not only in a narrow specialty but across multiple areas as well [60]. Changing demographic factors, such as population ageing, and the management of complex, multidisciplinary conditions are compelling the medical profession to adapt [60]. In order to support the global transformation of medical education, an increasing number of experts and organizations favour competency-based medical education (CBME). CBME is an approach also supported by the International Competency-Based Medical Education (ICBME) collaborators, with the aim of improving medical education worldwide [61]. Hungarian medical education is also increasingly adapting to international trends. In recent years, it has taken significant steps in the introduction of simulation-based education. The use of 3D printing, virtual reality, and augmented reality enables students to practise in realistic clinical settings, which is relevant from the perspectives of both patient safety and skills transfer [62]. The globalization of medicine and the international expansion of medical education increase the importance of accreditation systems and quality assurance efforts. The strategic partnership between the WHO and the WFME, together with the guidelines they have developed, plays an important role in shaping educational standards aimed at the global improvement of quality in medical education and the transparency of accreditation [63].

#### 4.2.2. Digital and Data Competencies (Data Literacy) as a New Basic Requirement

A key human prerequisite of the digital transition is that professionals should be able to interpret structured health data, use interoperable systems, and comply with the basic principles of data protection and cyber security [64]. For healthcare professionals, particularly nurses and physicians, it has become increasingly important to acquire the technical, ethical, and communication skills that enable them to cope with the above requirements. The 2023 international spring school Information in Healthcare—From Data to Knowledge highlighted that educational programmes of this kind enhance the capabilities required for data-driven decision-making, particularly in the areas of problem-solving, interprofessional collaboration, and critical thinking [64]. The lack of these competencies may represent one of the hidden bottlenecks of digitalization and, accordingly, curricular integration of data literacy is justified.

#### 4.2.3. Cultural Competence and Communication in Globalized Care

Ongoing changes associated with globalization and migration have reshaped the demographic landscape and created complex challenges for healthcare provision. Linguistic and cultural differences may generate barriers at organizational, structural, and clinical levels, and cultural competence interventions have been developed to mitigate these barriers [65]. These include the targeted recruitment of minorities into healthcare professions, the provision of interpreting services, the development of culturally and linguistically appropriate patient education materials, and intercultural training for healthcare professionals [65]. Research indicates that linguistic and cultural barriers may lead to health inequalities among patients with limited language proficiency. Collaboration with trained medical interpreters also appears as a legal requirement in several systems; however, provider training in this area is often lacking or inconsistent [66]. In order to ensure effective communication, it is essential that providers understand the role of interpreters, the good practices of working with them, and the ethical challenges involved [66]. At the same time, the effectiveness of cultural competence development cannot always be assessed unequivocally because of methodological heterogeneity. Long-term, attitude-shaping programmes (e.g., Program to Enhance Relational and Communication Skills-PERCS) indicate promising results, but their generalisability may be limited [67]. The conclusion here, too, is that the effects of globalization are not homogeneous: the outcomes of training and interventions depend strongly on the local institutional context and on the quality of implementation.

#### 4.2.4. Research Partnerships as a Competency-Creating Ecosystem

In the era of globalization, research collaborations aimed at addressing global health challenges are playing an increasingly important role. Outcome-oriented and cost-effective research models make it possible to develop interventions that may directly improve the well-being of individuals and communities [68]. Partnerships based on the involvement of stakeholders (policymakers, clinicians, and patients) may facilitate the practical utilisation of research findings, while also feeding training and competency development needs back into the system [69]. In order to improve the quality of healthcare research, there is a growing need for transparent, reproducible, and ethical research practices. In this respect, professionals working in clinical practice may also assume roles within informal or network-based collaborations [70]. This is important from the perspective of competencies, because critical thinking, data use, collaboration, and the responsible communication of results are becoming everyday expectations in the modern healthcare industry.

The globalization of human resources presents a dual challenge. On the one hand, the retention and fair distribution of the workforce, and, on the other, the rapid adaptation of competencies (digital and data literacy, intercultural communication, and interprofessional collaboration) is becoming a key factor. Sustainable responses therefore require both policy instruments (retention, ethical recruitment, and data-driven planning) and educational innovation (CBME, simulation, data competencies, and cultural competence) [53,61,64].

## 5. Digitalization, AI, and Data Governance

The interconnection of globalization and digitalization in the healthcare industry is creating new channels of care (telemedicine, remote monitoring), new operational logics (platform-based systems and interoperability), as well as new opportunities for decision support and automation (artificial intelligence, AI). At the same time, data governance (data quality, access regulation, responsibility, and transparency), together with data protection and cyber security, is becoming increasingly important, as the volume and value of health data are growing rapidly. The main application areas of AI and digitalization, together with their principal benefits and risks, are summarized in Table 2.

### 5.1. Digital Care Channels: Telemedicine and Remote Monitoring

Telemedicine—including synchronous (real-time), asynchronous (time-shifted), as well as mobile and home-monitoring systems—is playing an increasingly important role in reducing inequalities in access, particularly among geographically or socially disadvantaged populations [71]. The significance of telemedicine for the healthcare industry lies in the fact that the service can be “extended” beyond physical infrastructure, thereby reducing barriers to access and supporting the follow-up of chronic patients as well as the effectiveness of preventive programmes [71].

At the same time, the introduction of digital care is not merely a technological issue. It also has organizational, quality assurance, and human competency requirements, particularly where telemedicine becomes part of routine care. Most of the studies examined here emphasise that the expansion of digital health technologies opens up new dimensions in the efficiency of healthcare systems, while also raising ethical, data security, and operational challenges. The protection of patient data, the transparent use of algorithms, and equal access to technological tools are fundamental conditions of a sustainable and equitable digital health ecosystem [72].

It should also be emphasised that technological tools, particularly AI-based diagnostic and predictive solutions, may represent progress not only in terms of cost-efficiency, but may also contribute to the spread of personalised medicine. At the same time, AI-based systems require ethical scrutiny, particularly with regard to algorithmic bias, questions of decision-making responsibility, and the issue of who owns and benefits from the resulting health data assets [14,73]. The practical ecosystem of telemedicine is also being expanded by digital medical technologies. Wearable sensors, smart medication-dispensing solutions, and remote monitoring systems create new opportunities for the monitoring of chronic diseases and prevention through real-time data collection [74,75]. The resulting real-world data may support clinical decision-making and precision medicine, while at the same time reinforcing questions of data quality, data security, and responsibility [76].

### 5.2. Platformisation, Interoperability, and the Organization of Care

The operation of healthcare systems is under increasing pressure from persistent workforce shortages, further aggravated by low pay, growing workloads, and the migration of professionals. These factors not only endanger the quality of direct patient care but also constitute a major obstacle to the digital transition of healthcare [77].

The functioning of platform-based healthcare systems is increasingly determined by interoperability and the capacity to standardise data transmission, enabling data exchange and real-time access between different providers. Such systems are not merely IT developments but signal a transformation in the logic of care. Platform-level coordination becomes possible in relation to patient pathway management, access control, documentation, e-prescriptions, and other functions [78]. The aim of modern e-health solutions is to improve accessibility and make healthcare services more efficient. Their operation, however, requires not only advanced technological infrastructure, but also a well-prepared and available workforce. Owing to technological backwardness, many healthcare institutions are unable to exploit the opportunities offered by such systems, particularly where their everyday use would be required [78].

At the same time, structural differences arising from unequal access to digital infrastructure and knowledge become more pronounced. The digital literacy of older age groups, the limitations affecting the technological access of marginalised communities, and the lack of data protection and user trust are all barriers to which development policy must provide targeted responses [79,80]. This issue may affect older people in particular, as they increasingly require safe healthcare services that are also accessible from home. Telemedicine and digital access to prescriptions could provide them with a convenient and effective solution; however, the sustainable operation of such systems is possible only if adequate support is ensured on both the technological and human sides [80]. International experience makes it clear that technological advancement alone is not sufficient for successful digitalization: the effectiveness of digital healthcare systems is closely linked to transparent data governance, the digital training of professionals, and the level of social trust [79,81].

### 5.3. AI Applications: Decision Support, Automation, and Personalisation

The accelerating spread of AI and machine learning (ML) in the healthcare industry is opening up new areas of application, particularly in diagnostics, patient monitoring, care organization, and administrative automation [82,83]. The complexity of AI solutions ranges widely, from rule-based automation to deep learning models. From the perspective of industry benefit, the key requirement is that models should be validated, deployable, and auditable [8,14]. ML is one of the principal methodological families within AI, learning patterns from large volumes of data and providing prediction or decision support without explicit rule-based programming [83]. Artificial intelligence and machine learning have opened up new horizons in diagnostics and personalised medicine. The application of AI enables the rapid processing of genetic and clinical data, thereby supporting more targeted therapies and more accurate diagnoses [84,85]. The concept of personalised medicine has long been an enduring aspiration in medical science and has gained new momentum with the emergence of AI; however, at the same time, it may also entail the risks of distorted data interpretation and unequal access [14,86]. The proper application of AI may confront both developers and users with questions such as the selection of appropriate protocols, the identification of relevant variables, and the determination of how models may be integrated into clinical workflows [11]. Topol (2019) emphasises that technological developments can constitute genuine breakthroughs only if they become embedded in clinical practice and remain balanced with the requirements of human decision-making, patient safety, and transparency [8]. More recent literature similarly suggests that the real-world clinical benefit of AI in diagnostic and decision support applications depends on robust evaluation and appropriate regulatory oversight [87,88].

As shown in Table 2, AI and digitalization affect diagnostics, patient monitoring, care coordination, personalized medicine, and cybersecurity in different ways, but across these areas they consistently combine potential efficiency and quality gains with ethical, data protection, and liability-related challenges [8,14].

### 5.4. Data Governance, Global Databases, and Cyber Security

The data-driven nature of digital health is making the creation of increasing numbers of structured, large-scale databases necessary. The use of open-source software is of particular importance in this field, as it facilitates international research collaboration, may reduce the costs of scientific projects, and may contribute to improving the efficiency of healthcare delivery [89]. The spread of wearable sensors and wireless body area networks (WBANs) is revolutionising the collection and transmission of health data [90]. These data may support the early detection of diseases, improve diagnostic accuracy, and enhance treatment effectiveness. At the same time, however, the storage and transmission of sensitive information also entail significant security risks [91]. If unauthorised parties gain access to such data, they may even alter them, which may lead to erroneous decisions and patient safety risks [91]. Given the sensitivity of health information, the use of reliable encryption procedures is of key importance, particularly in defence against both active and passive attacks [90].

One promising direction in the use of global databases and networked data is federated learning, which allows multiple institutions to train models collaboratively without transferring raw patient data to a central location, thereby potentially offering a compromise between utility and data protection [75]. At the same time, this approach is not an “automatic” solution either as issues of data quality, model transparency, and risk management remain relevant here as well [72,75]. Technological progress has also brought new solutions to healthcare in the areas of access control and traceability. The combination of blockchain-based access control and hybrid deep learning techniques may enable the development of digital health systems that strengthen data protection and auditability, while also supporting cooperation among providers [92].

In summary, a key condition for the success of the digital health ecosystem is that AI- and platform-based solutions should operate within a secure, transparent, and responsibly governed data environment. The integrated use of global health databases and AI may play a central role in establishing more efficient and data-driven care, but only if issues of data protection, cyber security, model validation, and accountability are placed within manageable frameworks [14,33,85].

## 6. Ethics, Law, and Public Policy

Alongside technological acceleration and global interconnectedness, ethical and legal frameworks are not merely “supplements”, but prerequisites for the social acceptability of innovation and for equitable access.

### 6.1. Global Governance and National Implementation

Global health ethics is a relatively new concept that describes the issues of moral values and dilemmas arising in healthcare. In a broader sense, global health ethics is a normative project characterized by the challenge of responding to global health threats [93]. In parallel with globalization, the strengthening of global health security entails a number of ethical and legal challenges, particularly in relation to access to vaccines, data protection, and international cooperation. Health equity lies at the centre of the global approach to sustainable development, especially within the framework of the United Nations Sustainable Development Goals. The Global Alliance for Vaccines and Immunization (GAVI), the WHO, and the World Trade Organization (WTO) are key actors in this process, while national organizations also bear significant responsibility for implementation at the local level [25]. GAVI seeks not only to increase the availability of vaccines, but also, through its Ethics, Risk and Compliance Office, places emphasis on adherence to ethical standards. At the organizational level, the Ethics, Risk and Compliance Office ensures compliance with ethical guidelines, the management of risks, and the maintenance of regulatory compliance, thereby promoting transparency and accountability within the organization [94].

The WHO places particular emphasis on ensuring equitable access to health technologies. The organization stresses that everyone has the right to access safe and affordable medicines and vaccines, which is fundamental to the realization of the right to health. In addition, the WHO actively participates in global health coordination, for example through the COVID-19 Vaccines Global Access (COVAX) initiative, which aims to ensure the fair distribution of vaccines worldwide [95]. The role of the WTO in securing access to health technologies is manifested primarily through the framework of intellectual property rights and trade rules. The WTO cooperates with the WHO and the World Intellectual Property Organization (WIPO) in order to promote access to medical technologies, while also taking into account the protection of intellectual property rights [96]. National health organizations are responsible for implementing global guidelines at the local level. This includes ensuring data protection, adherence to ethical standards, and the application of legal regulations. The development and enforcement of national legal frameworks are essential to strengthening global health security and to ensuring equity, reciprocity, and social justice [97].

### 6.2. Intellectual Property, Patents, and Access

One of the central ethical and legal issues in global health innovation is patenting. The rapid development of new medicines and therapies, such as mRNA-based vaccines, has sharpened the dilemma between the protection of intellectual property and open access. International trade rules provide the framework for patent rights; however, at the same time, public health considerations periodically bring more flexible access arrangements to the fore. In addition, the pharmaceutical industry is under increasing pressure from external factors such as revenue losses resulting from patent expiry, healthcare systems striving for cost-efficiency, and tightening regulatory expectations [98]. Alongside corporate innovation strategies, these processes also reinforce the issue of social legitimacy as patent protection may stimulate innovation, but it may also hinder access if compensatory mechanisms are absent. While such regulations encourage innovation, they may also obstruct access to life-saving products in lower-income countries where flexible, solidarity-based patent-sharing mechanisms are not available. A good example is the amendment of Thailand’s patent law, after which little attention was paid to the impact of the change on medicine prices and accessibility, particularly among vulnerable groups [99]. Patent protection therefore influences not only the direction of innovation but also has a direct effect on the availability of therapies and on public health justice [24]. A major challenge for the future will be to develop patent and access arrangements that strike a balance between incentivising innovation and ensuring fair global access [24,99].

### 6.3. Crisis Governance, Pandemics, and Global Coordination

Globalization has accelerated the spread of infectious diseases and highlighted that, during pandemics, not only immediate response but also the development of long-term, sustainable health policy measures is of critical importance [25]. The COVID-19 pandemic clearly demonstrated how rapidly a regional health problem can become a global crisis. International mobility, the circulation of goods, and the complexity of supply chains enable the rapid spread of viruses [4]. Although the speed of vaccine development offered hope for containment, it also generated new challenges in relation to mass production, logistics, and equitable distribution. These processes made it clear that trust, transparent communication, and the allocation of supply capacities play a key role in the success of pandemic management [26,32].

Preparedness for pandemics therefore also has political and justice-related dimensions, requiring a reconsideration of global inequalities. A pandemic treaty or a more coordinated mechanism can be effective only if the countries concerned are given a voice in shaping regulation, and if international instruments take account of regional specificities and resource asymmetries [100]. The lessons of the COVID-19 pandemic have also shown that, alongside response, greater emphasis must be placed on prevention, particularly in the context of zoonotic diseases. An integrated approach that addresses public health, biodiversity, food security, and trade dimensions together may point towards a more sustainable and morally justifiable direction [101].

### 6.4. Ethical Dilemmas: Institutions, AI, and Data

Ethical approaches increasingly examine whether it is reasonable to evaluate organizations and their structures from an ethical perspective as well. Rather than judging only the behaviour of individuals as ethical or unethical, this approach implies that the functioning of healthcare systems and institutions as a whole, including their formal and informal relationships, also requires ethical evaluation [102]. For this reason, adherence to ethical norms is not merely a legal matter, but also a social expectation that influences the acceptance of technologies, the patient–provider relationship, and public trust [102]. Compliance with ethical standards, going beyond legal conformity, is closely related to quality, since good care is not only effective and safe, but also just, transparent, and respectful of patients’ dignity [103]. The use of health data, the role of AI algorithms in decision-making, and the protection of personal health information are all areas that require greater transparency and ethical governance [14,104]. The “four principles plus scope” approach—a bioethical analytical model based on the prima facie principles of respect for autonomy, beneficence, non-maleficence, and justice, as well as on the reflective examination of their scope of application—offers a simple and widely applicable framework for everyday ethical dilemmas [105]. In AI-related settings, this framework is complemented by the need to institutionalise accountability, auditability, and bias mitigation, particularly where technology affects clinical decisions or resource allocation.

### 6.5. Physical and Digital Security: Resilience and “One Health”

The vulnerability of global healthcare systems becomes particularly acute in times of crisis. Shortages of raw materials, transport disruptions, and the vulnerability of digital systems during pandemics have all highlighted the importance of security of supply. The One Health approach, building on the interconnections between human, animal, and environmental health, offers a framework for addressing the complex challenges of global health security [106]. In order to increase the resilience of healthcare systems, it is essential to protect digital infrastructure, apply international security standards, and manage supply risks in advance. The Joint External Evaluation (JEE) tool introduced within the framework of the International Health Regulations (IHR) represents an important milestone in strengthening global health security, particularly by placing emphasis on the assessment of legal frameworks [97]. The development of the legal component of this tool is relevant because the capacities required for crisis management (data sharing, the legitimacy of measures, and cooperation) often depend on legal and institutional conditions [97]. Strengthening primary care, together with integrating the One Health approach, provides an opportunity to enhance prevention and preparedness. The design and financing of global healthcare systems must be aligned with the aims of this approach in order to create healthcare systems that are sustainable, resilient, and accessible to all [25]. The ethical, legal, and public policy dimensions of globalization play a structuring role in the healthcare industry. The balance between innovation and access (patents and public health), the legitimacy and equity of crisis governance, and the transparency and accountability of AI- and data-driven operations together determine the extent to which the benefits of globalization can be realised and its risks can be controlled [14,25].

## 7. Sustainability and Climate

Globalization and the climate crisis are shaping the healthcare industry in mutually reinforcing ways. Extreme weather events, air pollution, and the environmental burdens of supply chains are simultaneously increasing morbidity and intensifying the operational risks faced by healthcare systems. The chapter examines (1) the health consequences of climate change and the need for system adaptation, (2) the public health and cost implications of air pollution, and (3) pathways for reducing the ecological footprint of the healthcare industry. Together, climate impacts and decarbonisation pressures create a “dual transition” in which the healthcare industry must become both more resilient and less carbon intensive.

### 7.1. Climate Change and Health Consequences

Extreme weather and climate events are posing an increasing threat to human health and well-being. Climate change is one of the greatest global challenges, and its health consequences are becoming ever more evident [107].

Climate change not only increases physical burdens (e.g., heatwaves and extreme events), but also places growing strain on mental health, and, furthermore, uncertainty, loss, and crises may have particularly disproportionate effects on vulnerable groups [108]. Environmental stress, economic hardship, and social vulnerability may together exacerbate mental health outcomes. This heterogeneity also means that the effects of climate adaptation and public health interventions may vary across countries and regions [108]. The 2023 *Lancet Countdown* report clearly demonstrates that the relationship between health and climate change is no longer a theoretical issue, but an urgent reality. Healthcare systems must adapt to increasing climate risks, while climate action also offers new, proactive opportunities to improve health. The report’s central message is that climate policy decisions materially shape public health outcomes [109].

The required health response focuses on three major challenges: promoting measures aimed at reducing carbon emissions and improving health; building climate-resilient and low-carbon healthcare systems; and implementing public health interventions against a range of climate-related risks [109]. Accordingly, the healthcare sector must simultaneously (1) adapt (e.g., through heatwave plans, capacity planning, and the targeted protection of vulnerable groups) and (2) reduce its own emissions, because the sustainability of its operation will increasingly depend on climate exposure in the future [109,110]. The health burdens of climate change and the need for system adaptation do not arise in a homogeneous way, therefore, rather than applying one-size-fits-all solutions, adaptive strategies based on local risk profiles are warranted [107,109]. This inequality is also supported by the systematised review of Naser et al., which found that low- and middle-income countries are among the regions most exposed to the health consequences of climate change, while accounting for only 14% of global CO_2_ emissions [111].

### 7.2. Air Pollution, Urbanisation, and Health Costs

Deteriorating air quality is closely linked to climate change and to the industrial, transport, and energy use characteristics of urban environments. As a result of global warming, dust and pollen exposure may increase, posing particular risks for those suffering from respiratory diseases. Globalised economic restructuring (for example, industrial transfers) may in some regions lead to adverse air-quality and health outcomes [112]. All this represents a significant risk, since certain pollutants, such as nitrogen dioxide (NO_2_), ozone (O_3_), and particulate matter (PM_10_), may be associated with increased morbidity and rising healthcare expenditure [113]. Taking these effects into account is of key importance for the healthcare sector, because industrial decisions and economic globalization indirectly affect air quality and, consequently, healthcare costs as well [112,113]. Global healthcare systems are paying increasing attention to the prevention of deteriorating population health through air quality monitoring and the integration of environmental data. During periods of peak pollution, reducing exposure (e.g., through alert systems and targeted guidance) may contribute to mitigating risks. Although, at the individual level, the relationship between symptoms and exposure may be complex, at the population level improvements in air quality typically result in health gains and potentially lower costs [114]. Together with climate risks, air pollution constitutes a common intersection of prevention and system planning, from which it follows that emission reduction is simultaneously a climate protection and a public health intervention [113,114].

### 7.3. The Ecological Footprint of the Healthcare Industry and Decarbonisation

The climate crisis requires urgent decarbonisation measures, and the healthcare sector also bears substantial responsibility for reducing its carbon footprint. Although traditional “green” initiatives, such as recycling and improving energy efficiency, are important steps, they are often not sufficient in themselves to achieve the necessary targets [110]. The ecological footprint of the healthcare sector is considerable: energy consumption, waste generation, and the environmental burdens of supply chains all contribute to environmental pressure. Alongside the environmental burdens associated with globalised procurement and long supply chains, challenges of quality control and transparency may also arise. Therefore, it is justified to link procurement and quality assurance with sustainability objectives [115]. The pharmaceutical industry may make a significant contribution to the greenhouse gas emissions of the healthcare sector, thereby intensifying climate change. Although several large companies have committed themselves to emission reduction targets—including carbon neutrality or net zero emissions—the extent of implementation varies, particularly with regard to emissions arising in the supply chain [115]. The optimisation of manufacturing, distribution, energy use, and water consumption play a key role on the path towards sustainability. The industry has considerable potential to reduce its climate impacts; however, greater transparency, more standardised reporting, and innovative solutions are needed [115,116].

Recognising all this, global healthcare systems offer an opportunity to advance green healthcare. In order to create the sustainable healthcare system of the future, the sector requires a fundamental long-term redesign that takes social and environmental impacts into account [117]. The more rapid diffusion of new technologies and procedures may contribute to the adoption of environmentally friendly solutions, such as energy-efficient infrastructure, well-designed digital documentation, waste reduction, and circular procurement [116,117]. The sustainability transition is of strategic importance because climate exposure and volatility in energy markets may directly affect both the continuity of care and cost structures [110]. It can be concluded that the decarbonisation of the healthcare industry is not merely a matter of “green” reputation. It is also relevant from the perspectives of patient safety, security of supply, and long-term financial sustainability. Effective approaches are typically based on a combination of (1) energy and infrastructure efficiency, (2) the greening of procurement and supply chains (3) the reduction of waste and material flows, and (4) transparent measurement and reporting [110,115,117].

Climate and sustainability in the healthcare industry thus constitute both a field of risk and a field of innovation. Climate-related harms increase pressure on care systems, while emission reduction and adaptation encourage a form of systemic modernisation that may support more resilient, more efficient, and more sustainable operation in the longer term [109,110].

## 8. Cultural Channels, Complementary and Alternative Medicine

Through the cultural channels of globalization, not only technologies and services, but also health concepts and therapeutic practices are spreading. One area in which this is particularly evident is complementary and alternative medicine (CAM), which appears in the healthcare industry both as a matter of consumer preference (health-conscious lifestyles and a holistic approach) and as a regulatory and epistemological challenge (evidence base, quality, patient safety, and authorisation).

### 8.1. Complementary and Alternative Medicine (CAM) in the Context of Globalization

As a result of globalization, interest in health-conscious lifestyles and non-conventional forms of treatment has increased across several regions. The literature associates the presence of CAM in part with the diversification of public preferences and the growing emphasis on wellbeing [118]. This trend may encourage healthcare systems to engage with CAM within clearly defined frameworks (e.g., patient information, risk communication, and integrative care pathways), while ensuring that the principles of patient safety and quality assurance are not compromised. CAM is an umbrella term, encompassing, for example, mind–body practices (such as yoga and meditation), herbal products and dietary supplements, as well as therapeutic systems linked to particular traditions. In some cases, religiosity and spirituality may support coping, subjective well-being, and adaptation to treatment. However, from the perspective of the healthcare industry, it is particularly important to foster realistic expectations and to distinguish claims that are supported by evidence. Among alternative treatments, certain practices, such as acupuncture, may also appear in some indications as complementary elements within Western care protocols, particularly in the management of pain and stress. Regional specificities (e.g., balneological traditions), together with globalization, may also support new markets and health tourism channels; however, this does not alter the fact that the assessment of clinical utility and patient safety remains a key issue.

Herbal products and dietary supplements are popular worldwide, reflecting growing demand for health-conscious lifestyles and for “natural” solutions. However, the quality and safety of these products require particular attention, as inadequately controlled or contaminated products may pose health risks. Questions of regulation and authorisation are therefore of central importance for the sustainability of the industry and for consumer protection [119]. The holistic approach, which understands health not only in physical but also in mental and social dimensions, is gaining increasing prominence in public discourse and in the range of services offered. At the same time, the social appeal of integrative approaches is not equivalent to proof of clinical effectiveness, thus, accordingly, the healthcare industry must treat claims that “support well-being” and those that “promise therapeutic effect” in a differentiated manner [120].

### 8.2. Evidence Base, Quality, and Regulation

A central issue in the integration of CAM into the healthcare industry is evidence-based practice and patient safety. While some methods may play a supportive role in certain indications, for others evidence of effectiveness remains insufficient, and risks may be undercommunicated. For this reason, validation, quality control, and accurate information are not merely scientific matters, but also regulatory and ethical ones. In the field of CAM, too, globalization accelerates the spread of knowledge and products, which may simultaneously increase access and market supply, while also intensifying risks (quality heterogeneity, lack of standardisation, interactions with medicines, and misleading marketing). Accordingly, regulatory responses must place particular emphasis on clarifying product categories and claims (e.g., dietary supplement vs. medicine; a “well-being” claim vs. a therapeutic claim), as well as on market surveillance and consumer protection. Digitalization creates new opportunities for the dissemination of CAM-related information and for supporting patient pathways, but it also increases the risks of disinformation and misleading health claims, therefore, the role of platform regulation, transparent communication, and health literacy becomes increasingly important [121]. It may be concluded that CAM is a visible phenomenon of the healthcare industry through the cultural channels of globalization, signalling the diversification of both demand and supply, and may be linked to preventive and well-being trends. At the system level, however, the main challenge lies in ensuring evidence-based validation, quality assurance, patient safety, and accurate risk communication. The role and acceptance of CAM differ from country to country; therefore, policy responses should follow an approach that is adapted to local culture and institutional settings, while remaining centred on patient safety [118,120].

## 9. Conclusions

The effects of globalization on the healthcare industry may be understood as a network of interrelated phenomena, and the thematic areas reviewed here represent different manifestations of the same systemic process. The findings of this study are consistent with the main conclusions of the literature, according to which global integration is not merely an economic category, but also a catalyst for social, technological, and cultural change in healthcare systems. The reviewed literature most consistently supports certain recurring patterns, such as supply-chain vulnerability, workforce imbalances, and the growing importance of data governance. At the same time, the interpretation of these patterns within a broader globalization framework should be understood as reflecting the conceptual synthesis of this review. The expansion of international clinical research, the spread of AI-based diagnostic and decision-support systems, and the cross-border flow of health data all illustrate how globalization permeates multiple levels of healthcare—from primary care and pharmaceutical development to health policy decision-making. The direct effects identified in this study, such as technology transfer, the vulnerability of supply chains, and the dynamics of service mobility (health tourism), suggest that globalization may simultaneously represent both an opportunity and a systemic risk. Taken together, these findings point to recurring trade-offs across channels, particularly between efficiency and resilience, innovation and security, and expanded access and equity. The COVID-19 pandemic demonstrated that the global health infrastructure is fragmented at several points, and that rapid technological progress is not necessarily accompanied by global health equity. This is illustrated by inequalities in access and by the consequences of global workforce flows, which may pose enduring challenges, particularly in low- and middle-income countries.

The findings of the study also indicate that artificial intelligence and digitalization should not be interpreted merely as technological innovations, but as developments capable of transforming the patient–provider relationship, the norms governing the use of health data, and the social dimensions of access to care. This makes it necessary to establish an interdisciplinary regulatory and governance framework that takes into account issues of data protection, transparency, accountability, and algorithmic bias. The clinical and organizational embedding of technology is likely to be sustainable only if digital infrastructure, human competencies (particularly data competencies), and legally and ethically coherent regulation develop in parallel.

The study further highlights that globalization is not a homogeneous process: the responsiveness of healthcare systems varies considerably across countries and regions. The tension between locality and global norms is evident in the standardization of healthcare protocols, in questions of security of supply, and in the balance between national sovereignty and international coordination. The guidelines of the WHO and other international organizations reflect an intention towards harmonisation; however, the outcomes of implementation may differ because of variations in local institutional capacities, financing environments, and levels of social trust. On this basis, the study suggests that greater emphasis should be placed on localised adaptation and on flexible, learning-oriented models of global governance.

Sustainability and climate-related risks, together with globalization, create a new type of adaptive pressure for the healthcare industry. The health burdens of climate change and the operational risks faced by healthcare systems (for example, adaptation to extreme events and dependencies related to energy and supply) are likely to intensify in the future, while reducing the ecological footprint of the healthcare sector is becoming strategically significant. Resilience and decarbonisation are, therefore, increasingly interconnected areas of concern.

In summary, the impact of globalization on the healthcare industry is not merely an economic or technological issue, but also one of social justice, access to health, and sustainable healthcare systems. From a health system perspective, the findings of this study point to four practical priorities that may be particularly relevant for policymakers, healthcare providers, regulators, and industry actors. First, supply vulnerabilities may be reduced through supplier diversification, strategic stockpiling, and, where feasible, stronger regional capacities. Second, workforce strategies should place greater emphasis on retention, competency development, and responsible international recruitment. Third, the expansion of digitalization and artificial intelligence should be supported by coordinated digital infrastructure, clear data management rules, cybersecurity safeguards, and well-defined accountability frameworks. Fourth, climate adaptation and sustainability considerations should be integrated into health system planning to support the continuity of care and long-term system resilience. One of the principal challenges for future health policy will be to harness the benefits of globalization—innovation, knowledge sharing, and expanded access—while at the same time purposefully reducing vulnerabilities (in supply chains, workforce shortages, and data security) and leaving room for solutions adapted to local circumstances.

The narrative nature of the review, the heterogeneity of the literature, and the absence of formal quality appraisal represent limitations of the review; therefore, the findings should be interpreted cautiously and with awareness of possible selection bias. Future research could refine or challenge these conclusions by examining these trade-offs comparatively across different health system contexts and by generating stronger evidence on how governance arrangements shape resilience, equity, and implementation capacity. The final lesson of this study, therefore, is that managing globalization in the healthcare industry requires an adaptive, value-based, and multi-level coordinated approach, while accepting that outcomes and impacts will inevitably differ across countries and regions.

## Figures and Tables

**Table 1:** Impact channel–consequence matrix: main channels of globalization and key healthcare industry outcomes.

Main Channels of Globalization	Access	Cost Efficiency	Quality and Patient Safety	Equity	Resilience	Innovation Dynamics	Risks
Pharmaceuticals, clinical development, and regulation	faster access	R&D efficiency	regulatory quality	unequal access	regulatory coordination	drug development, MRCTs	ethical, regulatory
Supply chains and resilience	product availability	economies of scale	supply disruptions	import dependence	diversification, stockpiling	logistics innovation	shortages, bottlenecks
Service mobility (health tourism)	cross-border access	lower cost, shorter waiting times	continuity of care	uneven benefits	-	service competition	infections, follow-up gaps
Human resources and competencies	workforce capacity	workforce shortages	competency base	brain drain	retention, training	education, upskilling	burnout, dependency
Digitalization, AI, and data governance	digital access	automation gains	decision support	digital divide	cyber resilience	AI, personalization	bias, data breaches
Ethics, law, and public policy	access frameworks	regulatory clarity	legitimacy, safety	justice, reciprocity	crisis governance	responsible adoption	weak implementation
Sustainability and climate	vulnerability of access	energy and system costs	environmental health burdens	vulnerable groups affected	adaptive capacity	green transition	extreme events, air pollution
Cultural channels/CAM	broader choice	-	variable evidence base	context- dependent uptake	-	service diversification	misinformation, weak standardization

Note: The matrix illustrates the main relationships identified in the literature review. R&D—Research and Development; MRCT—Multi-Regional Clinical Trial.

**Table 2:** Comparative Summary of AI and Digitalization in the Healthcare Industry.

Area	The Role of AI/Digitalization	Benefits	Risks/Challenges
Diagnostics	Analysis and prediction based on imaging and clinical data	Earlier and more accurate detection	False positives/negatives, accountability, and validation
Patient Monitoring	Analysis of data from wearable devices	Continuous monitoring and self-management	Data protection and device reliability
Care Coordination/ Administration	Support for documentation, triage, and patient pathway optimization	Improved efficiency and reduced burden	Interoperability, bias, and auditability
Personalized Medicine	Patterns in genetic/epigenetic data	Targeted therapy and better response	Discrimination and bias in data interpretation
Cybersecurity/Governance	Access control, logging, and anomaly detection	Enhanced control and support for compliance	New attack surfaces and increased complexity

Source: Author’s own compilation (2025), based on the literature reviewed.

## Data Availability

No new data were created or analyzed in this study. Data sharing is not applicable to this article.
